# Miso (Fermented Soybean Paste) Suppresses Visceral Fat Accumulation in Mice, Especially in Combination with Exercise

**DOI:** 10.3390/nu11030560

**Published:** 2019-03-06

**Authors:** Ran Okouchi, Yuto Sakanoi, Tsuyoshi Tsuduki

**Affiliations:** Laboratory of Food and Biomolecular Science, Graduate School of Agriculture, Tohoku University, Sendai 981-8555, Japan; orchid5416@gmail.com (R.O.); s-noi.nod@ezweb.ne.jp (Y.S.)

**Keywords:** fermented soybean paste, visceral fat accumulation, miso, anti-obesity, exercise

## Abstract

We investigated whether the difference in miso consumption between the Japanese diets of 1975 and 2010 has influenced the observed increase in diet-induced obesity. To recreate the 2010 and 1975 Japanese high-fat diets with the corresponding proportions of miso, freeze-dried miso was added to high-fat mouse feed at 1.6% and 2.6%, respectively. When 5-week-old male Institute of Cancer Research (ICR) mice were provided each of these diets ad libitum for 8 weeks, it was found that the white adipose tissue weight and adipocyte area were lower in mice receiving the 1975 diet than in those receiving the 2010 diet. Therefore, high miso consumption is one reason why the 1975 Japanese diet tended to not lead to obesity. Next, the combined effects of treadmill exercise and miso consumption were investigated. The mice were divided into three groups, which were provided either a high-fat diet (group C), a high-fat diet with exercise (group C + E), or a miso-supplemented high-fat diet with exercise (group M + E) for 8 weeks. In this experiment, the white adipose tissue weight and adipocyte area in group M + E were lower than in group C. When the mRNA expression of lipid metabolism-associated genes in adipose tissue was measured, we found that expression of *Hsl* (lipase, hormone sensitive), which is involved in lipolysis, and *Pparγ* (peroxisome proliferator activated receptor gamma), which regulates adipocyte differentiation upstream of *Hsl*, was increased in group M + E. These results clearly demonstrated that lipid accumulation in the adipose tissues is suppressed by miso consumption in combination with exercise.

## 1. Introduction

Compared with other countries worldwide, Japan has one of the longest life expectancies [1]. We previously investigated the Japanese diet, which is a lifestyle component unique to Japan, as a factor favoring longevity. The Japanese diet has changed since the end of World War II; accordingly, we evaluated the effects of the diets of each era on the health of mice [2]. Specifically, the diets of 2005, 1990, 1975, and 1960, according to food consumption surveys, were recreated by mixing 21 foods typically consumed during 1 week and freeze-drying and pulverizing the mixture. Subsequently, the diets were provided to mice. The 1975 diet, with a high miso content, had more pronounced anti-obesity effects than the current Japanese diet [2].

Consumption of miso by Japanese people has decreased continuously since 1975, with the consumption rate in 2010 approximately half that in 1975 [3,4]. Miso is prepared by mixing soybeans, malted rice, wheat or barley, and salt and allowing the mixture to ferment and mature. The principal ingredient, soybeans, contains large quantities of soybean-specific proteins (glycinin and β-conglycinin), lipids rich in polyunsaturated fatty acids, vitamin E, lecithin, saponin, and isoflavones (genistein, daidzein, daidzin, and glycitein) [5,6,7]. Therefore, soybeans are a functional food, and fermentation thereof results in a high nutrient content that includes amino acids and isoflavones. Miso has previously been reported to effectively prevent lifestyle-related diseases such as cancer, hypertension, and hypercholesterolemia [5,6,7]. With its antioxidant activities, miso may play a role in preventing aging due to oxidative stress [8]. However, the anti-obesity effects of miso have been rarely investigated.

Obesity leads to metabolic disorders such as dyslipidemia and type 2 diabetes and is linked to life-threatening conditions such as arteriosclerosis; thus, prevention of obesity is critical [9,10]. Most previous research has focused on single food components in miso and not the functionality of miso itself in the diet. Moreover, most research has focused on the efficacy of miso components at a very high level, with almost no investigation of their efficacy at concentrations present in a real diet.

In addition to diet, exercise is effective for the prevention of obesity. Exercise increases the energy demand of skeletal muscles, which leads to energy generation by mitochondria, with sugars and lipids as substrates [11]. Along with substrates present in tissues, muscles generate energy using glucose and fatty acids extracted from the bloodstream [12,13]. Furthermore, lipolysis is promoted in adipose tissues, forming fatty acids, whereas in the liver, glucose is formed by gluconeogenesis; these substances are subsequently transferred to the blood and then transported to the muscles [12,13]. Exercise thus promotes energy consumption. Food components such as catechin and chlorogenic acid are known to affect this pathway and thus potentiate the effects of exercise [14,15,16]. Therefore, foods containing such components are considered to be highly beneficial for maintaining health.

In this context, the present study focused on miso and the effects of its consumption on the development of obesity. Two test diets containing miso at levels similar to those in ordinary Japanese diets in 1975 and 2010 based on food consumption surveys were prepared. The effects of these test diets on obesity in mice were then investigated. Subsequently, we investigated whether the combination of exercise and miso consumption potentiates the anti-obesity effects of exercise.

## 2. Materials and Methods

### 2.1. Preparation of Test Diets

Miso (fermented soybean paste) provided by Marukome Co., Ltd. was first freeze-dried (FD-550R, Tokyo Rika Kikai Co., Ltd., Tokyo, Japan). According to the National Health and Nutrition Survey, the daily miso intake in 1975 and 2010 was 20.8 g and 10.8 g, respectively [3,4]. Given the moisture content of miso (38.5%), these amounts correspond to 12.8 g and 6.6 g, respectively. The ratio of miso (*w*/*w*) intake to total daily food intake (excluding water) based on the National Health and Nutrition Survey in 1975 or 2010 was calculated as 2.6% or 1.6% (dry weight %), respectively. Test diets was prepared by adding freeze-dried miso at each calculated proportion to a high-fat-modified AIN-93G diet (Table 1). In Experiment 1, a diet containing 2.6% of freeze-dried miso was used as the 1975 miso diet, and a diet containing 1.6% of freeze-dried miso served as the 2010 miso diet. In order to standardize the general nutritional composition and salinity of the test diets, miso-replacing material (produced to match the nutritional composition of freeze-dried miso, Supplemental Table S1) was added to the 2010 miso diet at 1.0%; the total content of freeze-dried miso and miso-replacing material was set at 2.6%. In addition, a diet containing 2.6% of miso-replacing material was prepared as a control diet. The miso-replacing material consisted of casein as protein, soybean oil as fat, corn starch as carbohydrates, NaCl as a salt equivalent, and cellulose as a bulking agent (Supplemental Table S1). In Experiment 2, a diet containing 2.6% of miso-replacing material served as a control diet, and a diet containing 2.6% of freeze-dried miso was used as the miso diet (Table 1). Energy was calculated using the modified Atwater method (4 kcal of protein, 9 kcal of fat, and 4 kcal of carbohydrates per g) [17]. The energy per 100 g of each test diet was 491 kcal.

### 2.2. Animals

All animal procedures were performed in accordance with the Animal Experiment Guidelines of Tohoku University and approved by the Animal Use Committee at Tohoku University (2016AgA-009) [18,19]. Male Institute of Cancer Research (ICR) mice (4 weeks old) were obtained from Clea Japan (Tokyo, Japan). The mice were provided ad libitum access to their respective diet and distilled water in a temperature- and humidity-controlled room with a 12/12-h light/dark cycle. In Experiment 1, after acclimatization to a CE-2 powder diet for 1 week, 30 mice were randomly divided into three groups (two cages with five mice per cage) that were fed the control diet, 2010 miso diet, or 1975 miso diet (control, 2010 miso, or 1975 miso group, respectively) for 8 weeks. In Experiment 2, during the acclimatization period of 1 week, 40 mice were fed a CE-2 powder diet and performed running exercise under preliminary test conditions. At 5 weeks of age, all mice were divided into four groups of 10 mice each (two cages with five mice per cage), such that the average body weights of the groups were nearly equal. The groups were fed the control diet without exercise (group C), miso diet without exercise (group M), control diet with exercise (group C + E), and miso diet with exercise (group M + E) for 8 weeks. Exercise was performed using a treadmill (MK-680, Muromachi Kikai, Tokyo, Japan) as previously described [20]. Movement on the treadmill was carried out from 17:00 until 20:00, just before the start of the dark period, during which mice are most active. The exercise conditions during the acclimatization period included 18 m/min, 10 min/day, and 3 days/week. After 5 weeks of age, the C + E and M + E groups performed running exercise under conditions of 20 m/min, 50 min/day, and 5 days/week for 8 weeks. Because all mice were able to complete the exercise regimen, all mice were subjected to the same routine. In both Experiments 1 and 2, the mice were weighed, and blood samples were collected by decapitation at 13 weeks of age after a 12-h fast. To obtain serum, the blood samples were centrifuged (900× *g*, 5 °C, 15 min). The brain, heart, lung, liver, spleen, pancreas, kidney, and white adipose tissues (epididymal, mesenteric, and perinephric adipose tissues) were removed and weighed. Serum and organs were stored at −80 °C until use.

### 2.3. Histological Analysis

For histological analysis, liver and epididymal adipose tissue were fixed in 10% formalin and embedded in paraffin [21]. Vertical sections (5 μm) were cut, mounted on a glass slide, stained with hematoxylin and eosin (HE), and observed using a microscope (BZ-9000, Keyence, Osaka, Japan). The mean area of adipocytes was calculated in each group [21].

### 2.4. mRNA Expression Analysis

For real-time quantitative reverse transcriptase polymerase chain reaction (qRT-PCR), total RNA was isolated from the liver using an RNeasy Mini Kit (Qiagen, Valencia, CA, USA) [22], eluted with 40 μL of RNase-free water, and stored at −80 °C until use. The mRNA levels of acetyl-coenzyme A carboxylase beta (*Acc*); acyl-coenzyme A oxidase 1, palmitoyl (*Aco*); patatin-like phospholipase domain containing 2 (*Atgl*); actin, beta (*β-actin*); cytochrome P450, family 7, subfamily a, polypeptide 1 (*Cyp7a1*); fatty acid synthase (*Fasn*); glucose-6-phosphate dehydrogenase X-linked (*G6pdx*); 3-hydroxy-3-methylglutaryl-coenzyme A reductase (*Hmgcr*); lipase, hormone sensitive (*Hsl*); malic enzyme (*Me*); peroxisome proliferator activated receptor alpha (*Pparα*); peroxisome proliferator activated receptor gamma (*Pparγ*); and sterol regulatory element binding transcription factor 1 (*Srebp1c*) in the liver were determined with the Thermal Cycler Dice Real Time System® (Takara Bio, Otsu, Japan). This system allows real-time quantitative detection of PCR products by measuring the increase in fluorescence caused by binding of SYBR green to double-stranded DNA [23]. In brief, cDNA was generated from total RNA in the liver using Prime Script® RT Master Mix (Perfect Real Time, Takara Bio, Otsu, Japan). The cDNA was subjected to PCR amplification using SYBR® Premix Ex TaqTM (Perfect Real Time, Takara Bio) and gene-specific primers for *Acc*, *Aco*, *Atgl*, *β-actin*, *Cyp7a1*, *Fasn*, *G6pdx*, *Hmgcr*, *Hsl*, *Me*, *Pparα*, *Pparγ*, and *Srebp1c* (Supplemental Table S2). PCR amplification was performed with activation at 95 °C for 10 s, followed by 40 cycles at 95 °C for 5 s (denaturation) and 60 °C for 31 s (extension), and dissociation at 95 °C for 15 s, 60 °C for 30 s, and 95 °C for 15 s for each gene. Melting curve analysis was performed following each reaction to confirm the presence of only a single reaction product. The threshold cycle (Ct) value, representing the PCR cycle at which an increase in reporter fluorescence above a baseline signal can first be detected, was also determined for each reaction. The *β-actin* level in test samples was used to normalize gene expression levels.

### 2.5. Serum and Liver Biochemical Analyses

Biochemical analyses of serum and liver samples were performed as described previously [24,25]. Serum and liver triacylglycerol (TG), total cholesterol (TC), and serum phospholipid (PL) and glucose were measured using commercial enzyme kits (Wako Pure Chemical, Osaka, Japan). Serum insulin was determined using an enzyme-linked immunosorbent assay (ELISA) kit (Morinaga Institute of Biological Science, Yokohama, Japan). Liver PLs were measured using the procedure described by Rouser et al. [26]. A microplate reader (Infinite F200, Tecan Japan Co., Ltd., Kanagawa, Japan) was used for the absorbance measurement.

### 2.6. Statistical Analysis

Results are expressed as the mean ± standard error of the mean (SE). In Experiment 1, data were analyzed by one-way analysis of variance (ANOVA) followed by the Tukey–Kramer post hoc test, if appropriate. In Experiment 2, the effect of the miso diet and the exercise showed the effect of the diet, the exercise, and the interaction between miso and exercise among four groups by using two-way ANOVA. For parameters that showed a significant difference, one-way ANOVA with a Tukey–Kramer post hoc test was used among all groups. All analyses were performed with a significance level of α = 0.05 (*p* < 0.05) using IBM-SPSS statistics version 26 (IBM SPSS Inc., Chicago, IL, USA).

## 3. Results

### 3.1. Experiment 1

**Figure 1:**
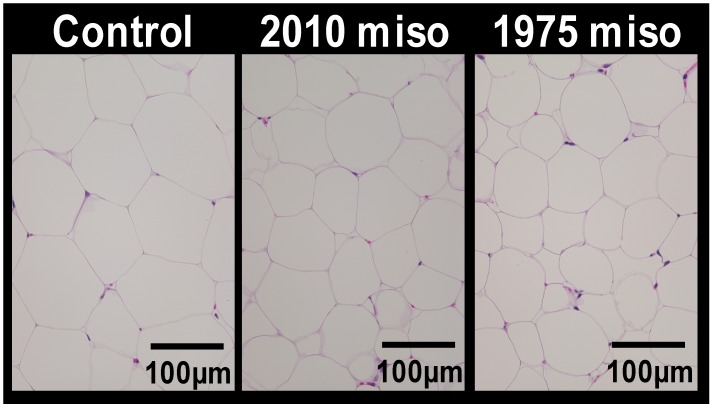
Effect of dietary intake of miso on adipocyte hypertrophy. Hematoxylin-eosin (HE) staining of epididymal adipose tissue sections from respective mice of each group (scale bar = 100 µm).

No significant differences in body weight and food or energy intake were found between the three study groups (Table 2). The 1975 miso group had significantly greater heart and kidney weights relative to body weight than the control and 2010 miso groups, but no significant differences between the groups were found in the absolute weights of these organs or the brain, liver, lungs, pancreas, or spleen. The weights of the three white adipose tissues were significantly lower in the 1975 miso group compared to other groups. Because differences in white adipose tissue weights were found, tissue sections were stained with HE, and the adipocyte sizes were observed, with the adipocytes appearing smaller in the 1975 miso group than in the control and 2010 miso group (Figure 1). Quantitative assessment of the adipocyte sizes showed a similar tendency; the adipocyte area in the 1975 miso group was significantly less than that in the control group (*p* < 0.05), with the area ratio in the three groups measured as follows: control group, 1.00 ± 0.11; 2010 miso group, 0.86 ± 0.11; and 1975 miso group, 0.74 ± 0.09. On the basis of these findings, the 1975 miso diet was considered to have anti-obesity effects.

### 3.2. Growth Parameters (Experiment 2)

**Figure 2:**
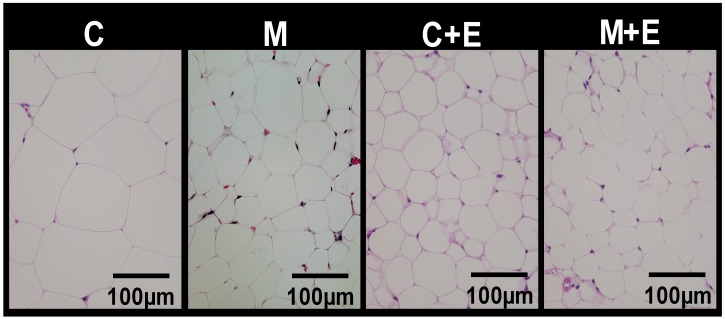
Effect of exercise and dietary intake of miso on adipocyte hypertrophy. Hematoxylin-eosin staining of epididymal adipose tissue sections from respective mice of each group (scale bar = 100 µm).

Growth parameters were measured to investigate how differences in miso and exercise influence body weight and organ weights (Table 3). There was no significant difference in initial body weight, final body weight, food intake and energy intake in the respective pairs of groups. Brain and kidney weights were increased by exercise load, but with no significant differences among the 4 groups. There was no significant difference in heart, liver, lung, pancreas, or spleen weight in the respective pairs of groups. The white adipose tissue weights were decreased by the miso diet and exercise. Epididymal and mesenteric adipose tissue weights were significantly lower in the C + E and M + E groups than in the C group, significantly lower in the M + E group compared to the M group, and the lowest in the M + E group. Perinephric adipose tissue weight was significantly lower in the C + E and M + E groups than in the C group, significantly lower in the M + E group compared to the M and C + E groups, and the lowest in the M+E group. Because differences in white adipose tissue weight were found, tissue sections were stained with HE, and adipocyte sizes were observed, revealing smaller adipocytes in the groups that were subjected to miso or exercise (Figure 2). Quantitative assessment of the adipocyte sizes showed a similar tendency, with the adipocyte area decreased by the miso diet and exercise; the adipocyte area ratio in the 4 groups were as follows: group C, 1.00 ± 0.05; group M, 0.80 ± 0.06; group C + E, 0.71 ± 0.06; and group M + E, 0.65 ± 0.04. The adipocyte area ratio was significantly lower in the M, C + E and M + E groups than in the C group, significantly lower in the M + E group compared to the M group, and the lowest in the M + E group. These findings demonstrate that miso consumption in combination with exercise potentiated the suppressive effect on fat accumulation in white adipose tissue, in comparison with exercise alone. With respect to skeletal muscle weights, the weights of the gastrocnemius, quadriceps femoris and tibialis anterior muscles was increased by the exercise and soleus muscle weight was increased by the miso diet. The weights of the gastrocnemius and quadriceps femoris muscles were significantly higher in the M + E group than in the C group, and the highest in the M + E group, but there were no significant differences in the weights of the soleus and tibialis anterior muscles among the 4 groups.

### 3.3. mRNA Expression Level in Epididymal Adipose Tissue (Experiment 2)

To investigate the mechanism by which the combination of exercise and miso consumption reduces fat accumulation in adipose tissues, expression of lipid metabolism-associated genes in adipose tissue surrounding the testes was measured by qRT-PCR (Table 4). When mRNA associated with fatty acid synthesis was measured, *Acc* and *G6pdx* mRNA levels were increased by exercise load. *Acc* mRNA level was significantly higher in the C + E group than in the C group, and significantly higher in the C + E and M + E groups compared to the M group. *G6pdx* mRNA level was significantly higher in the C + E group compared to the M group. There was no significant difference in *Fasn* and *Me* mRNA levels in the respective pairs of groups. The mRNA level of *Srebp1c*, which is a transcription factor for genes associated with fatty acid synthesis, was increased by exercise load, and significantly higher in the C + E group than in the C and M groups. The mRNA level of *Hsl*, a gene involved in intra-adipocytic lipolysis, was increased by the miso diet, exercise, and interaction between the diet and exercise, and significantly higher in the C + E and M + E groups than in the C group, significantly higher in the M + E group compared to the M and C + E groups, and the highest in the M + E group. The mRNA level of *Atgl*, another gene involved in lipolysis, was increased by exercise, and significantly higher in the C + E and M + E groups than in the C group, significantly higher in the M + E group compared to the M group, and the highest in the M + E group. And, the trend was similar to that of *Hsl*. *Pparγ*, which is involved in adipocyte differentiation and miniaturization, was increased by the miso diet and exercise, and significantly higher in the C + E and M + E groups than in the C and M groups, significantly higher in the M + E group compared to the C + E groups, and the highest in the M + E group. These findings clearly demonstrated that miso consumption in combination with exercise potentiates adipocyte lipolysis and adipocyte miniaturization, in comparison with exercise alone.

### 3.4. Biochemical Parameters in Serum and Liver (Experiment 2)

**Figure 3:**
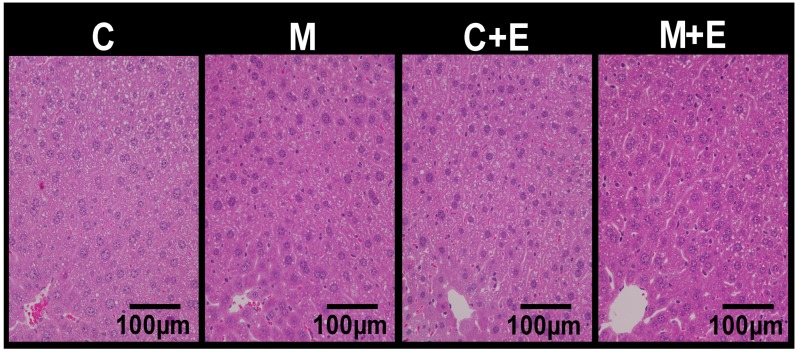
Effect of exercise and dietary intake of miso on liver. Hematoxylin-eosin staining of liver sections from respective mice of each group (scale bar = 100 µm).

The effects of exercise and miso consumption on serum lipid levels and glucose metabolism were investigated (Table 5). The serum glucose concentration was decreased by the miso diet and exercise, and significantly lower in the M + E group than in the C and M groups, and the lowest in the M + E group. No significant differences between the 4 groups were found in other serum parameters. The effects of exercise and miso consumption on fat accumulation in the liver were next investigated. The hepatic triacylglycerol (TG) and total cholesterol (TC) concentrations were decreased by the miso diet and exercise, and significantly lower in the M + E group than in the C groups, and the lowest in the M + E group. The phospholipid (PL) concentrations showed no significant differences between the 4 groups. Because differences in fat accumulation in the liver were found, tissue sections were stained with HE, and the cell morphology was observed (Figure 3). Fewer intra-adipocytic lipid droplets were observed in group M+E than groups C and C + E, and fat accumulation in the liver appeared to be suppressed. Thus, miso consumption in combination with exercise appeared to potentiate the suppressive effects on fat accumulation in the liver, in comparison with exercise alone.

### 3.5. mRNA Expression Level in Liver (Experiment 2)

To investigate the mechanism by which fat accumulation in the liver is reduced by exercise and miso consumption, hepatic expression of genes associated with lipid metabolism was measured by qRT-PCR (Table 6). Regarding genes associated with fatty acid synthesis, the *Acc* mRNA level was decreased by miso diet and exercise, significantly lower in the M + E group than in the C and C + E groups, and the lowest in the M + E group. *G6pdx* mRNA level was increased by exercise load, but with no significant differences among the 4 groups. There were no significant differences in levels of *Fasn*, *Me*, or *Srebp1c* mRNA in the respective pairs of groups. The mRNA level of *Aco*, a gene involved in fatty acid catabolism, was decreased by the miso diet, exercise, and interaction between the diet and exercise, and significantly lower in the M+E group than in the C, M, and C + M groups, whereas *Pparα*, which regulates fatty acid catabolism, showed no significant difference between the 4 groups. The mRNA level of *Hmgcr*, which is associated with cholesterol synthesis, was decreased by miso diet, significantly lower in the M + E group than in the C and C + E groups, and the lowest in the M + E group. The mRNA level of *Cyp7a1*, which is associated with cholesterol catabolism, showed no significant difference between the 4 groups. As a result, miso consumption in combination with exercise suppressed fatty acid and cholesterol synthesis in the liver.

## 4. Discussion

In Experiment 1, we investigated the effects of consumption of miso at quantities similar to those in typical Japanese diets in the years 1975 and 2010 on obesity in mice. When mice were provided the 2010 and 1975 test diets with miso contents of 1.6% and 2.6%, respectively, the mice provided the 1975 miso diet had significantly lower white adipose tissue weight than those provided the 2010 miso diet. In addition, adipocyte size was significantly lower in the 1975 group than the control group. These findings revealed that high miso consumption suppresses fat accumulation in white adipose tissue and thus has anti-obesity effects.

Soybeans contain large quantities of soybean-specific proteins (glycinin and β-conglycinin), lipids rich in polyunsaturated fatty acids, vitamin E, lecithin, saponin, and isoflavones (genistein, daidzein, daidzin, and glycitein). In particular, soybean proteins and isoflavones have been shown to reduce visceral fat accumulation in rats and mice with genetic or diet-induced obesity [27,28]. The suppressive effect of miso on visceral fat accumulation observed in this study suggested that these miso components function in an additive or synergistic manner. In addition, this effect was achieved at a level common to an actual human diet, rather than at artificially high levels.

Miso was a staple of the 1975 Japanese diet, which had high anti-obesity effects. This study showed that miso suppressed obesity induced by a high-fat diet and, therefore, may be partially responsible for the anti-obesity effects of the 1975 Japanese diet. The difference in miso content between the 1975 and 2010 test diets was only approximately 1 g per 100 g—equivalent to one to two bowls of miso soup in a human diet—yet different effects on the development of obesity were found. We recently showed that consumption of natto, another fermented soybean product, or the 1975 Japanese diet markedly changes the intestinal microflora [29,30]. Changes in the intestinal microflora are known to affect the development of numerous diseases, and it is therefore possible that the results of the present study are attributable to changes in the intestinal microflora.

Experiment 2 was conducted to determine whether the known anti-obesity effects of exercise are potentiated by combination with miso consumption. We confirmed that miso consumption in combination with exercise potentiates suppression of fat accumulation in white adipose tissue and the liver, in comparison with exercise or miso intake alone. In addition, adipocyte size was decreased in this group. To elucidate the mechanism underlying these effects, the expression of genes associated with lipid metabolism was measured in the white adipose tissue, and it was found that *Pparγ* mRNA was increased by exercise and further increased by miso consumption. *Pparγ* is a transcription regulatory factor that is expressed at high levels in adipocytes and regulates adipocyte differentiation and proliferation [31]. In a study in which rats fed high-fat diets were administered *Pparγ* agonists, the number of hypertrophic adipocytes was reduced, and the number of miniaturized adipocytes was increased [32]. Therefore, *Pparγ* is considered to play important roles in suppressing excessive adipocyte hypertrophy and ensuring normal adipocyte size [33]. Moreover, macrophage infiltration and inflammatory cytokine secretion increase in connection with adipocyte hypertrophy and, therefore, suppression of adipocyte hypertrophy is important for preventing obesity and insulin resistance [34]. Furthermore, owing to its influence on metabolism in various organs, prevention of adipocyte hypertrophy is critical. In the present study, exercise and miso consumption suppressed adipocyte hypertrophy, conferring a positive effect on the whole body. Moreover, this combination significantly reduced blood glucose. Exercise-induced activation of *Pparγ* in adipose tissue promotes lipolysis, mediated by *Hsl* and *Atgl* [35,36]. In the present study, high expression of *Hsl* and *Atgl* mRNA was observed as a result of exercise and miso consumption. Exercise-induced, *Hsl*- and *Atgl*-mediated lipolysis plays an important role in the systemic supply of fatty acids [37,38] and is linked to suppression of fat accumulation in adipose tissues. This is not a transient effect occurring only at the time of exercise, as long-term effects have been confirmed when exercise is continued. Therefore, *Pparγ* activation due to exercise and miso consumption was considered to promote *Hsl*- and *Atgl*-mediated lipolysis, thereby reducing white adipose tissue weight.

Long-term, habitual exercise results in muscle fiber hypertrophy [39] and increased insulin sensitivity [40]. In the present study, skeletal muscle weight and glucose metabolism were increased by exercise, and miso consumption potentiated these effects. The exercise regimen implemented in this study was of moderate intensity, at 50% to 75% of maximal oxygen consumption [41,42], equivalent to gentle running by humans. At this exercise intensity, both carbohydrates and lipids are used as energy sources [43], and the routine was, therefore, effective for suppression of visceral fat accumulation.

Exercise alone had no significant effects on hepatic fat accumulation due to a high-fat diet, but this accumulation was significantly reduced by miso consumption in combination with exercise. Expression of *Acc* mRNA in the liver did not change with exercise alone, but it decreased significantly with miso consumption in combination with exercise. *Acc* is the rate-limiting factor for fatty acid synthesis and, under the condition of a high-fat diet, *Acc* suppression is known to suppress fatty liver development [44]. Consequently, the combination of exercise and miso consumption was considered to suppress lipid synthesis in the liver. Soybean proteins, which are contained in miso, suppress hepatic fat accumulation in genetically obese rats, and suppression of hepatic fatty acid synthesis has been reported as one factor involved in this process [45,46]. Similar effects have been reported with isoflavone [45,46]. In addition, exercise resulted in decreased expression of genes associated with fatty acid β-oxidation in the liver, but this was considered a physiological response occurring in conditions of decreased lipid levels. Based on the aforementioned observations, the effects of various functional components of miso along with exercise synergistically suppress fat accumulation in the liver.

## 5. Conclusions

Previous research revealed that individuals who consume a high amount of miso also tend to consume an abundance of vegetables, fish, and shellfish [2,12,47]. Therefore, habitual miso intake is expected to greatly improve dietary habits. In addition, with habitual exercise, achieving longevity and maintaining health are more likely. However, since this study is only the animal study, it is necessary to confirm the effect by a human intervention study in the future. We are planning to research about this in the future.

## Figures and Tables

**Table 1:** Composition of test diets (Experiment 1 and 2).

	Experiment 1			Experiment 2	
	Control Diet	2010 Miso Diet	1975 Miso Diet	Control Diet	Miso Diet
(g/100 g)					
Casein	19.48	19.48	19.48	19.48	19.48
Soybean oil	6.82	6.82	6.82	6.82	6.82
Lard	19.48	19.48	19.48	19.48	19.48
Cornstarch	19.24	19.24	19.24	19.24	19.24
α-Cornstarch	12.86	12.86	12.86	12.86	12.86
Sucrose	9.74	9.74	9.74	9.74	9.74
Cellulose	4.87	4.87	4.87	4.87	4.87
Mineral mix (AIN-93G-MX)	3.41	3.41	3.41	3.41	3.41
Vitamin mix (AIN-93-VX)	0.97	0.97	0.97	0.97	0.97
L-Cysteine	0.29	0.29	0.29	0.29	0.29
Choline bitartrate	0.24	0.24	0.24	0.24	0.24
tert-Butylhydroquinone	0.0014	0.0014	0.0014	0.0014	0.0014
Freeze-dried miso	0.00	1.60	2.60	0.00	2.60
Miso-replaced material ^1^	2.60	1.00	0.00	2.60	0.00
(kcal/100 g)					
Energy	491	491	491	491	491

^1^ Miso replaced material reproduced to match the nutritional composition of miso (Supplemental Table S1).

**Table 2:** Body weights, food intake and tissue weights (Experiment 1).

	Control	2010 Miso	1975 Miso
Initial body weight (g)	30.1 ± 0.5	30.1 ± 0.5	30.0 ± 0.5
Final body weight (g)	46.5 ± 1.5	46.6 ± 2.2	42.4 ± 1.4
Food intake (g/day)	3.60 ± 0.04	3.64 ± 0.06	3.64 ± 0.08
Energy intake (kcal/day)	17.7 ± 0.2	17.9 ± 0.3	17.9 ± 0.4
Tissue weight (g/100 g body weight)			
Brain	1.03 ± 0.04	1.03 ± 0.04	1.15 ± 0.04
Heart	0.41 ± 0.02 ^a^	0.41 ± 0.01 ^a^	0.48 ± 0.02 ^b^
Kidney	1.35 ± 0.04 ^a^	1.37 ± 0.05 ^a^	1.59 ± 0.04 ^b^
Liver	3.61 ± 0.07	3.60 ± 0.06	3.63 ± 0.10
Lung	0.63 ± 0.07	0.65 ± 0.06	0.55 ± 0.02
Pancreas	0.81 ± 0.04	0.80 ± 0.02	0.80 ± 0.03
Spleen	0.23 ± 0.02	0.22 ± 0.02	0.26 ± 0.02
White adipose tissue			
Epididymal	3.75 ± 0.32 ^b^	3.66 ± 0.38 ^b^	2.64 ± 0.18 ^a^
Mesenteric	1.69 ± 0.14 ^b^	1.49 ± 0.16 ^b^	0.99 ± 0.11 ^a^
Perinephric	2.22 ± 0.20 ^b^	2.07 ± 0.19 ^b^	1.23 ± 0.18 ^a^

Values are mean ± standard error (SE), *n* = 10. ^a,b^ Different superscript letters indicate significantly different means at *p* < 0.05.

**Table 3:** Body weights, food intake and tissue weights (Experiment 2).

	C	M	C + E	M + E	Interaction
Initial body weight (g)	30.0 ± 0.5	30.0 ± 0.5	30.0 ± 0.5	30.1 ± 0.5	
Final body weight (g)	42.5 ± 1.2	40.4 ± 1.0	39.2 ± 1.0	38.7 ± 1.2	
Food intake (g/day)	3.62 ± 0.09	3.62 ± 0.06	3.53 ± 0.10	3.45 ± 0.10	
Energy intake (kcal/day)	17.8 ± 0.4	17.8 ± 0.3	17.3 ± 0.5	16.9 ± 0.5	
Tissue weight (g/100 g body weight)					
Brain	1.13 ± 0.03	1.18 ± 0.04	1.24 ± 0.03	1.25 ± 0.03	E
Heart	0.51 ± 0.02	0.51 ± 0.01	0.52 ± 0.02	0.53 ± 0.02	
Kidney	1.61 ± 0.04	1.65 ± 0.05	1.74 ± 0.05	1.76 ± 0.05	E
Liver	3.67 ± 0.08	3.63 ± 0.10	3.56 ± 0.06	3.66 ± 0.10	
Lung	0.69 ± 0.06	0.70 ± 0.10	0.77 ± 0.06	0.79 ± 0.09	
Pancreas	0.84 ± 0.05	0.83 ± 0.03	0.84 ± 0.02	0.85 ± 0.04	
Spleen	0.27 ± 0.02	0.28 ± 0.02	0.32 ± 0.03	0.27 ± 0.02	
White adipose tissue					
Epididymal	3.59 ± 0.39 ^c^	2.84 ± 0.13 ^b,c^	2.38 ± 0.23 ^a,b^	1.52 ± 0.19 ^a^	M, E
Mesenteric	1.88 ± 0.16 ^c^	1.64 ± 0.13 ^b,c^	1.25 ± 0.14 ^a,b^	0.81 ± 0.11 ^a^	M, E
Perinephric	2.29 ± 0.16 ^c^	1.73 ± 0.18 ^b,c^	1.42 ± 0.15 ^b^	0.70 ± 0.11 ^a^	M, E
Skeletal muscle					
Gastrocnemius	0.87 ± 0.02 ^a^	0.89 ± 0.02 ^a,b^	0.92 ± 0.02 ^a,b^	0.95 ± 0.02 ^b^	E
Quadriceps	0.95 ± 0.03 ^a^	1.02 ± 0.05 ^a,b^	1.08 ± 0.03 ^a,b^	1.10 ± 0.03 ^b^	E
Soleus	0.11 ± 0.01	0.12 ± 0.01	0.11 ± 0.01	0.12 ± 0.01	M
Tibialis	0.33 ± 0.01	0.33 ± 0.01	0.35 ± 0.01	0.36 ± 0.01	E

Values are mean ± SE, *n* = 10. Because there were two factors of miso and exercise, statistical analysis was performed using two-way analysis of variance (ANOVA) and for items with significant differences one-way ANOVA was performed and followed by Tukey–Kramer’s test. ^a,b,c^ Different superscript letters indicate significantly different means at *p* < 0.05. Miso diet effect, exercise effect and interaction were described at the right side of the table: M, miso diet effect; E, exercise effect; M × E, interaction.

**Table 4:** mRNA expression level in epididymal adipose tissue (Experiment 2).

Gene Function	Gene Name	C	M	C + E	M + E	Interaction
Fatty acid synthesis	*Acc*	1.00 ± 0.15 ^a,b^	0.81 ± 0.14 ^a^	1.94 ± 0.27 ^c^	1.60 ± 0.23 ^b,c^	E
	*Fasn*	1.00 ± 0.16	0.99 ± 0.11	0.82 ± 0.12	0.74 ± 0.10	
	*Me*	1.00 ± 0.27	0.93 ± 0.23	0.88 ± 0.27	0.74 ± 0.24	
	*G6pdx*	1.00 ± 0.09 ^a,b^	0.83 ± 0.10 ^a^	1.22 ± 0.09 ^b^	1.12 ± 0.11 ^a,b^	E
	*Srebp1c*	1.00 ± 0.13 ^a^	0.81 ± 0.14 ^a^	1.68 ± 0.29 ^b^	1.22 ± 0.08 ^a,b^	E
Lipolysis	*Atgl*	1.00 ± 0.17 ^a^	1.22 ± 0.11 ^a,b^	1.95 ± 0.27 ^b,c^	2.61 ± 0.32 ^c^	E
	*Hsl*	1.00 ± 0.13 ^a^	1.23 ± 0.08 ^a,b^	1.73 ± 0.24 ^b^	2.91 ± 0.22 ^c^	M, E, M × E
Differentiation	*Pparγ*	1.00 ± 0.14 ^a^	1.24 ± 0.13 ^a^	2.04 ± 0.25 ^b^	3.07 ± 0.24 ^c^	M, E

Values are mean ± SE, *n* = 10. Because there were two factors of miso and exercise, statistical analysis was performed using two-way ANOVA and for items with significant differences one-way ANOVA was performed and followed by Tukey–Kramer’s test. ^a,b,c^ Different superscript letters indicate significantly different means at *p* < 0.05. Miso diet effect, exercise effect and interaction were described at the right side of the table: M, miso diet effect; E, exercise effect; M × E, interaction.

**Table 5:** Biochemical parameter of serum and liver (Experiment 2).

	C	M	C + E	M + E	Interaction
Serum					
	(mmol/L)				
TG	1.06 ± 0.07	1.01 ± 0.08	1.10 ± 0.12	0.93 ± 0.09	
TC	3.05 ± 0.21	2.81 ± 0.18	2.94 ± 0.09	2.77 ± 0.19	
PL	2.58 ± 0.05	2.51 ± 0.17	2.41 ± 0.14	2.46 ± 0.15	
Glucose	7.13 ± 0.42 ^b^	6.30 ± 0.19 ^b^	5.72 ± 0.63 ^a,b^	4.53 ± 0.22 ^a^	M, E
	(pmol/L)				
Insulin	36.2 ± 5.4	36.2 ± 5.4	30.1 ± 5.4	25.2 ± 2.7	
Liver					
	(µmol/g)				
TG	56.6 ± 6.7 ^b^	46.6 ± 3.9 ^b^	50.6 ± 6.4 ^b^	25.5 ± 2.5 ^a^	M, E
TC	20.5 ± 2.0 ^b^	19.4 ± 1.2 ^a,b^	19.0 ± 1.6 ^a,b^	14.4 ± 0.8 ^a^	M, E
PL	43.9 ± 1.1	43.8 ± 0.9	45.2 ± 0.6	44.8 ± 0.7	

Values are mean ± SE, *n* = 10. Because there were two factors of miso and exercise, statistical analysis was performed using two-way ANOVA and for items with significant differences one-way ANOVA was performed and followed by Tukey–Kramer’s test. ^a,b^ Different superscript letters indicate significantly different means at *p* < 0.05. Miso diet effect, exercise effect and interaction were described at the right side of the table: M, miso diet effect; E, exercise effect; M × E, interaction. TG, Triacylglycerol; TC, Total cholesterol; PL, phospholipid.

**Table 6:** mRNA expression level in liver (Experiment 2).

		C	M	C + E	M + E	Interaction
Fatty acid synthesis	*Acc*	1.00 ± 0.19 ^b^	0.77 ± 0.07 ^a,b^	0.85 ± 0.10 ^b^	0.39 ± 0.04 ^a^	M, E
	*Fasn*	1.00 ± 0.12	0.84 ± 0.10	1.05 ± 0.17	0.88 ± 0.09	
	*Me*	1.00 ± 0.14	1.25 ± 0.14	1.28 ± 0.12	1.14 ± 0.10	
	*G6pdx*	1.00 ± 0.02	1.07 ± 0.10	1.21 ± 0.09	1.24 ± 0.10	E
	*Srebp1c*	1.00 ± 0.11	0.86 ± 0.11	0.95 ± 0.04	1.07 ± 0.12	
β-oxidation	*Aco*	1.00 ± 0.09 ^b^	0.93 ± 0.07 ^b^	0.81 ± 0.07 ^b^	0.39 ± 0.05 ^a^	M, E, M × E
	*Pparα*	1.00 ± 0.07	1.11 ± 0.12	1.02 ± 0.08	0.96 ± 0.05	
Cholesterol synthesis	*Hmgcr*	1.00 ± 0.19 ^b^	0.63 ± 0.12 ^a,b^	0.92 ± 0.15 ^b^	0.39 ± 0.07 ^a^	M
Cholesterol catabolism	*Cyp7a1*	1.00 ± 0.11	1.24 ± 0.10	1.02 ± 0.13	1.08 ± 0.12	

Values are mean ± SE, *n* = 10. Because there were two factors of miso and exercise, statistical analysis was performed using two-way ANOVA and for items with significant differences one-way ANOVA was performed and followed by Tukey-Kramer’s test. ^a,b^ Different superscript letters indicate significantly different means at *p* < 0.05. Miso diet effect, exercise effect and interaction were described at the right side of the table: M, miso diet effect; E, exercise effect; M × E, interaction.

## Supplementary Materials

The following are available online at https://www.mdpi.com/2072-6643/11/3/560/s1, Table S1: Composition of Miso sample and replaced material, Table S2: Primer pairs used for the real time qRT-PCR analysis.
