# Supplementary material for: Miso (Fermented Soybean Paste) Suppresses Visceral Fat Accumulation in Mice, Especially in Combination with Exercise
Source: Nutrients. 2019 Mar 6;11(3):560. doi: 10.3390/nu11030560 (PMC6470805; doi:10.3390/nu11030560)
Supplement: Supplementary file 1 [file nutrients-11-00560-s001.pdf]

nutrients

MDPI

Article

Miso (Fermented Soybean Paste) Suppresses Visceral Fat Accumulation in Mice, Especially in Combination with Exercise

Ran Okouchi, Yuto Sakanoi and Tsuyoshi Tsuduki *

Laboratory of Food and Biomolecular Science, Graduate School of Agriculture, Tohoku University, Sendai 981-8555, Japan; orchid5416@gmail.com (R.O.); s-noi.nod@ezweb.ne.jp (Y.S.); tsudukit@tohoku.ac.jp (T.T.)

* Correspondence: tsudukit@tohoku.ac.jp; Tel.: +81-22-757-4392

Supplementary Materials

Table S1. Composition of Miso sample and replaced material.

Miso sample		Miso replaced material [1]	
	(g/100g)		(g/100g)
protein	16.6	casein	16.6
lipid	8.6	soybean oil	8.6
carbohydrate	47.7	corn starch	47.7
Salt equivalent	19.0	NaCl	19.0
other	8.1	cellulose	8.1
Energy (kcal/100g)	335	Energy (kcal/100g)	335

[1] Miso replaced material reproduced to match the nutritional composition of Miso.

Table 2. Primer pairs used for the real time qRT-PCR analysis.

Genbank ID	Gene name		Primer sequence (5′ to 3′)
NM_133904	*Acc*	Forward	CGCTCACCAACAGTAAGGTGG
		Reverse	GCTTGGCAGGGAGTTCCTC
NM_015729	*Aco*	Forward	TAACTTCCTCACTCGAAGCCA
		Reverse	CTGGGCGTAGGTGCCAATTA
NM_001163689	*Atgl*	Forward	CAACGCCACTCACATCTACGG
		Reverse	GGACACCTCAATAATGTTGGCAC
NM_007393	*β-actin*	Forward	GGCTGTATTCCCCTCCATCG
		Reverse	CCAGTTGGTAACAATGCCATGT
NM_007824	*Cyp7a1*	Forward	GGGATTGCTGTGGTAGTGAGC
		Reverse	GGTATGGAATCAACCCGTTGTC
NM_007988	*Fasn*	Forward	CCTGGATAGCATTCCGAACCTG
		Reverse	TTCACAGCCTGGGGTCATCTTTGC
NM_008062	*G6pdx*	Forward	TGGGTCCACCACTGCCACTTTTG
		Reverse	ATTGGGCTGCACACGGATGACCA
NM_008255	*Hmgcr*	Forward	AGCTTGCCCGAATTGTATGTG
		Reverse	TCTGTTGTGAACCATGTGACTTC
NM_001039507	*Hsl*	Forward	TTCTCCAAAGCACCTAGCCAA
		Reverse	TGTGGAAAACTAAGGGCTTGTTG
NM_001198933	*Me*	Forward	CCTCACCACTCGTGAGGTCAT
		Reverse	CGAAACGCCTCGAATGGT
NM_011144	*Pparα*	Forward	AGAGCCCCATCTGTCCTCTC
		Reverse	ACTGGTAGTCTGCAAAACCAAA
NM_011146	*Pparγ*	Forward	GGAAGACCACTCGCATTCCTT
		Reverse	TCGCACTTTGGTATTCTTGGAG
NM_011480	*Srebp1c*	Forward	GATGTGCGAACTGGACACAG
		Reverse	CATAGGGGGCGTCAAACAG

Acc, acetyl-Coenzyme A carboxylase beta; *Aco*, acyl-Coenzyme A oxidase 1, palmitoyl ; *Atgl*, patatin-like phospholipase domain containing 2; *β-actin*, actin, beta; *Cyp7a1*, cytochrome P450, family 7, subfamily a, polypeptide 1; *Fasn*, fatty acid synthase; *G6pdx*, glucose-6-phosphate dehydrogenase X-linked; *Hmgcr*, 3-hydroxy-3-methylglutaryl-Coenzyme A reductase; *Hsl*, lipase, hormone sensitive; *Me*, Malic enzyme; *Pparα*, peroxisome proliferator activated receptor alpha ; *Pparγ*, peroxisome proliferator activated receptor gamma; *Srebp1c*, sterol regulatory element binding transcription factor 1 sterol regulatory element binding transcription factor 1.
